# NEXCADE: Perturbation Analysis for Complex Networks

**DOI:** 10.1371/journal.pone.0041827

**Published:** 2012-08-03

**Authors:** Gitanjali Yadav, Suresh Babu

**Affiliations:** 1 Computational Biology Laboratory, National Institute of Plant Genome Research, New Delhi, India; 2 School of Human Ecology, Ambedkar University, New Delhi, India; Institut Pluridisciplinaire Hubert Curien, France

## Abstract

Recent advances in network theory have led to considerable progress in our understanding of complex real world systems and their behavior in response to external threats or fluctuations. Much of this research has been invigorated by demonstration of the ‘robust, yet fragile’ nature of cellular and large-scale systems transcending biology, sociology, and ecology, through application of the network theory to diverse interactions observed in nature such as plant-pollinator, seed-dispersal agent and host-parasite relationships. In this work, we report the development of NEXCADE, an automated and interactive program for inducing disturbances into complex systems defined by networks, focusing on the changes in global network topology and connectivity as a function of the perturbation. NEXCADE uses a graph theoretical approach to simulate perturbations in a user-defined manner, singly, in clusters, or sequentially. To demonstrate the promise it holds for broader adoption by the research community, we provide pre-simulated examples from diverse real-world networks including eukaryotic protein-protein interaction networks, fungal biochemical networks, a variety of ecological food webs in nature as well as social networks. NEXCADE not only enables network visualization at every step of the targeted attacks, but also allows risk assessment, i.e. identification of nodes critical for the robustness of the system of interest, in order to devise and implement context-based strategies for restructuring a network, or to achieve resilience against link or node failures. Source code and license for the software, designed to work on a Linux-based operating system (OS) can be downloaded at http://www.nipgr.res.in/nexcade_download.html. In addition, we have developed NEXCADE as an OS-independent online web server freely available to the scientific community without any login requirement at http://www.nipgr.res.in/nexcade.html.

## Introduction

Complex dynamical systems govern the patterns and processes observed across all domains of life, ranging from molecular frameworks within our cells to large-scale ecological communities, even globally interlinked social associations, transportation networks and internet communication [1], [2], [3]. Such systems are increasingly being conceptualized as interconnected networks using graph theory as a unifying language for exploration of a given entity in context of its structural or functional neighborhood [4], [5], [6]. This is an interdisciplinary approach that combines high throughput experimental techniques with computational mathematical analysis. In recent years, it has been successfully employed in almost all kinds of system-wide data exploration efforts for quantitatively defining the principles governing organizational complexities [7], [8]. Well documented applications of the network paradigm to systems as diverse as inter atomic chemical bonding networks [9], [10], viral infectome or human diseasome networks [11], [12], [13], co-authorship networks [14], and many others, highlight the success and efficacy of this method in providing insights towards a more complete understanding of the system.

Systems biology (or network science) is now witnessing a tremendous interest in the ‘robust, yet fragile’ nature of complex systems, arising from the recognition that they are not immune to attack or failure [15], [16], [17], [18]. Cellular malfunctions and diseases that often arise from perturbations in the intermolecular communication channels between bio-molecules [19], [20] or terrorist attacks that can instantly impair international air traffic and communication [21], have revealed the necessity and importance of predicting the behavior of a system in response to different kinds of disturbances. It has been observed that catastrophic changes in the overall state of a system can ultimately derive from its organization, or from linkages that may often be latent and unrecognized. Here-in lies the strength of computational systems biology and graph based mathematical tools which can enable prediction of global structural reorganizations upon perturbation.

Although perturbation analyses have now become routine exercises in both experimental and bioinformatics data interpretation, there is currently no automated mechanism of simulating the technique. Induced perturbations may be small, large, local, global, single, grouped, or sequential; they may be loss based or modifications of existing functionalities as in the outage of an interface in a power-grid network. For example, analysis of the yeast proteome network has shown that the likelihood of lethality upon node loss, (or the phenotypic consequence of a single gene deletion) is affected to a large extent by the topological position of its protein product in the interaction network [22]. Similarly, loss of an edge, as in case of disruption of hydrogen bonds by strong electrostatic repulsion is sufficient to destroy the stability of cross-beta network in amyloid fibrils [9]. Analysis of the *E.coli* metabolic network has shown that a non-hub node can also be vital to the stability of the network if it connects one or more key structural or functional modules [23]. The affects of paired perturbations can also be equally informative as single perturbations, such as in case of synthetic lethal interactions where loss of both nodes in a genetic network can be fatal to the cell [24], [25]. Extending the same concept, insights from the analyses of grouped perturbations can help in understanding the roles played by the nodes in that group, arising from modular functional units within the graph structure. In contrast to these real-world perturbation scenarios, sequential perturbations are studied more as ‘simulations’ to understand the possible affects of cascading disturbances on complex systems. Simulation of sequential perturbations is a standard technique employed in ecological network analyses, where the global biodiversity crisis and rapid population declines have galvanized investigations in the possible cascading affects of species extinctions and quantitative estimation of species loss [26], [27], [28]. This approach involves targeted removal of each entity from a given network in a sequential manner based on a specific attribute of the targeted node, most commonly, its degree or the number of links [29], [30], [31]. In all such analyses, the respective networks may show robust (perturbation independent) or non-robust (perturbation dependent) behavior in response to different perturbation. The outcomes of such studies provide insights into ‘network resilience’, i.e, the ability of a system to achieve fault tolerance against failures of its components [32], [33].

These examples illustrate the need for development of appropriate tools for analysis and modeling of perturbations in real world networks, since a large number of potential users do not have the requisite computational skills or mathematical background to carry out such analyses for their data. Accordingly, a broad range of academic and commercial platforms and tools are available for generic analyses, comparisons and visualization of networks and their properties. However, one of the major lacunae in this field is the assessment of network resilience or susceptibility, upon perturbation [34], [35]. Such a functional limitation becomes very important in view of the fact that this area is fast becoming one of the most prominent areas of network science, as also evident from the increasing numbers of publications dealing with perturbations and their affects [NCBI Pubmed Jan 2012 data]. However, in most cases, the perturbation analyses involve physics, mathematics and synthetic data whereas, it is equally important to focus on empirical real world data, since the architecture of complex biological, social and economic networks show topologies differing radically from random networks [2].

Based on our insights from an extensive analysis of the architecture of more than a hundred large publicly available real world networks and their responses under attack, we have developed NEXCADE, a program for simulation and analysis of perturbations in a complex system, and to monitor the altered system attributes at every step, in order to determine how associated perturbations are either generated or propagated from the previous event. Apart from an existing Cytoscape plugin that assesses the affects of protein abundance changes on protein-protein interaction (PPI) networks [35] from within Cytoscape [36], NEXCADE, to our knowledge, is the only software available to date that enables diverse kinds of perturbation analyses on all types of networks. We provide NEXCADE in two modes, an online web server for quick testing of the program’s capabilities and as a downloadable standalone unix package. The NEXCADE software is designed to automate the analysis of the vulnerability of networks based on the quantitative assessment of the impact of small or large-scale, static or dynamic perturbations. Despite the seeming differences between different types of real-world networks, we find that perturbations can affect these systems in very similar ways, since real world networks share several architectural properties especially scale free topology, high clustering coefficients, short average path lengths and greater than expected diameters [4]. NEXCADE would benefit users transcending varied disciplines; from a plant physiologist comparing gene regulatory networks across different species, or a biochemist searching for drug targets, to a restoration ecologist, or even a banker interested in identifying critical risk areas in a financial network.

## Analysis

### Network Concepts and Indicators

A graph is defined as a non empty set of nodes, a set of edges or links, and a map that assigns two nodes to each link [37]. We denote a network as a binary undirected graph G  =  (*V,E*) where V is the set of nodes (vertices) while E is the set of undirected edges (links) between two nodes if they are functionally linked to one another. Nodes of the network may represent genes, proteins, species or any entity of interest. In functional terms, an edge signifies relationships or ties or functional interaction between two nodes. Edges between a vertex and itself are not included. In graphical terms, each element of the set E is a pair of elements of set V. Although in many situations, links can be assigned a direction and a positive or negative weight to designate the strength of interaction, NEXCADE simplifies such graphs and uses only binary pair-wise connections for analyses. For a given network G, Network Size is denoted as S{G} and calculated as the total number of nodes in G. The Degree *k* of each node *i* is calculated as the total number of vertices adjacent to node *i*, and *k(i)  =  |N(i)|* where N is the neighborhood of node *i*, or the set of vertices adjacent to *i.* The density of the graph measures how many edges are in set E compared to the maximum possible number of edges between vertices in set V. For an undirected network that has no loop and can have at most |V| * (|V| − 1)/2 edges, the density is measured as 2 * |E|/(|V| * (|V| − 1)). The average degree of the network is K{G} =  *(sum(k(i))/S{G}),* where *k(i)* is the degree *k* of each node *i* as explained above, and S is the size of the network. The distance *d(i,j)* between two vertices *i* and *j* is the length of the shortest path from *i* to *j*, considering all possible paths in G from *i* to *j*. The distance between any node and itself is 0. If there is no path from *i* to *j* then *d(i,j)* is infinity.

### Input Format

For input, users can select between different kinds of undirected and un-weighted datasets for analysis, such as protein- protein interaction data, co-expression data, bipartite ecological webs of interactions between organisms, and social network data. In this manner, users are prompted at the outset to classify their data in order to delineate terms used thereafter, throughout the analyses. For example, a node may be a gene, protein or a species depending upon the type of network being studied. Similarly, an edge may be a relationship between two individuals in a social network or an interaction between two ORFs in a PPI network. Data is entered into NEXCADE in a simple and user-friendly format, as a list of interactions per line separated by a whitespace, which is then converted into graph format, such that each line of input defines an edge for the network that connects the node listed in the first column with the node listed in the second column. In this manner, information about network components and their interactions is read in as undirected and un-weighted. Loops if any, are removed, and each output line denotes two nodes that are connected to each other by an edge.

### Network Preprocessing & Visualization

In this step, each input graph is scanned for basic topological statistics including structural properties at the vertex, edge and network levels, using in-house Fortran programs and custom made shell scripts that incorporate the graphical capabilities of IGRAPH [38] within R CRAN (http://www.r-project.org/) for complex network research. Verification of whether a graph is connected is an essential preprocessing step. A graph that is fully connected has exactly one connected component, consisting of the whole graph. In disconnected graphs, each connected component of an undirected graph is a sub graph in which symmetric and transitive paths connect any two vertices to each other, and which is connected to no additional vertices. The number of connected components is an important topological invariant of a graph that plays a key role in the definition of graph toughness or robustness and we use this attribute to color the graph during visualization. The connected components of a graph are computed using breadth-first search, beginning at some vertex v and finding the entire connected component containing v (and no more) before completing. To find all the connected components of a graph, loops are run through its vertices, starting a new search whenever the loop reaches a vertex that has not already been included in a previously found connected component. Finally the network nodes are assigned colors based upon the connected component they belong to, and visualization of optimal component distribution is enabled using the fruchterman-reingold vertex layout algorithm [39].

For every vertex or node in the network, four topological centrality measures are calculated. These include the degree centrality k, betweenness centrality, closeness centrality and eigenvector centrality [40]. The vertex betweenness can roughly be defined by the number of geodesics (shortest paths) going through that vertex ‘*v’* and is measured as, *sum(G_ivj/G_ij, i! = j,i! = v,j! = v)* where G is the graph and *v* is the vertex in question, and ivj is the shortest path from *i* to *j* passing through *v*. The Closeness centrality roughly measures the number of steps required to access every other vertex from a given vertex. For a given vertex, this is defined by the inverse of the average length of the shortest paths to/from all the other vertices in the graph: *(|V|-1)/sum(d(v,i), i ! =  v)*. If there is no (directed) path between vertex v and i then the total number of vertices is used in the formula instead of the path length. Eigenvector centrality corresponds to the values of the first eigenvector of the graph adjacency matrix; which may, in turn, be interpreted as arising from a reciprocal process in which the centrality of each vertex is proportional to the sum of the centralities of other vertices that are directly connected to it. In general, vertices with high eigenvector centralities are those which are connected to many other vertices which are, in turn, connected to many others and so on [41].

### Perturbations of Network Components

In graphical terms, we define a perturbation as a random or targeted loss of one or more nodes or edges from a given network. Loss of a node indicates the deletion of an entity while the loss of an edge implies the destabilization of a function between two existing entities. The assumption is that each node in the network can function only of it has at least a single support link connecting it to another node in the network. NEXCADE employs the IGRAPH library at its backend to carry out each perturbation event while using a variety of shell scripts and R functions to compute and present topological affects and for plotting graphs. Each perturbation removes one or more nodes or links from the network and we find the fraction of nodes that remain functional at the end of the process. For example, if perturbation of an entity X causes another entity Y to lose its entire support link to the remaining sub-network, it (Y) is considered to become non-functional, or to have undergone ‘secondary extinction’ in association with entity X. In this manner, behavior of the network after each successive perturbation is monitored to measure robustness or susceptibility, in terms of the “cost” associated with each vertex removal [17], [26], which in turn may signify change in any of the local or global network properties, or additional perturbations generated by propagation of the previous event/s, such as secondary extinctions or associated co-extinctions as explained above. An additional and interesting dimension has been added to NEXCADE for assessing how a given network reacts to the random removal of any one node at a time. In this approach, all nodes are taken out and put back into the network, one at the time and topological properties are calculated and plotted across the removal of all the individual nodes while the network size remains constant as complete network minus one. These curves can than be compared across networks to assess how different networks behave upon random single perturbations. The cascading or ‘targeted’ perturbation approach involves simulations of random or ordered primary extinctions based on a given node property such as the number of links or ‘degree’. In summary, nodes of the input network are sorted and ranked by degree. Each of these nodes are then systematically removed in the sorted order, either from highest connected node to the least connected node or vice versa. For random cascades, all nodes are shuffled and then removed one after the other in a random sequence. Randomization can be repeated as many times as desired for comparative purposes. After every single node removal, the network is analyzed in terms of various properties described above. The reduced network is then used for carrying out removal of the next node in the list, followed by complete analysis, and so on. Finally, the structural integrity of the network is predicted for each loss sequence based on the threshold period for complete collapse, and the change in critical global topological attributes during the entire cascade are plotted as a function of the percentage of nodes perturbed. This method has been well established over the last decade and the response of the network in terms of resulting secondary extinctions or other network properties can be used to infer the significance of the node attribute being studied [29], [30], [31]. The sub-network remaining after each subsequent perturbation to the original input network can be visualized as described above. NEXCADE can also plot the complete outcomes of multiple perturbation cascade curves to enable comparative analysis of one extinction sequence with the others.

### Program Automation & Testing

**Figure 1:**
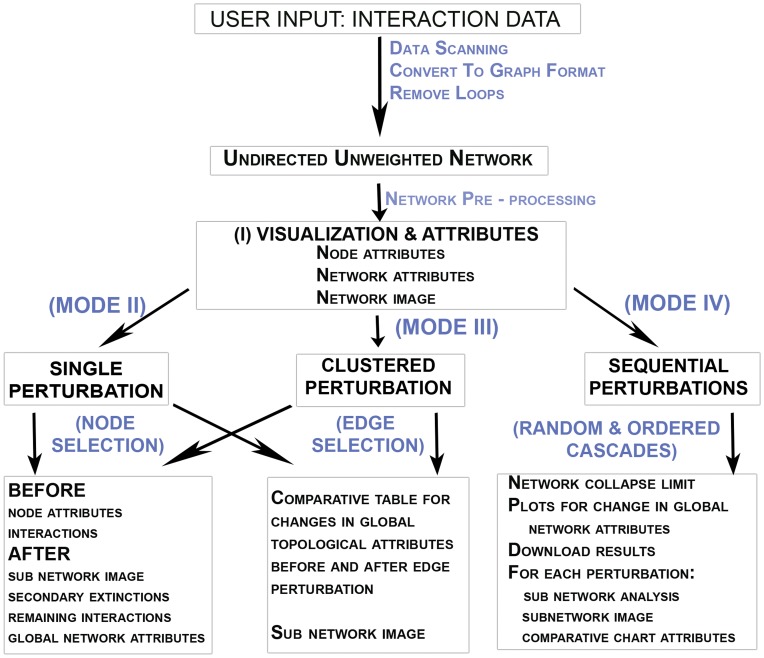
Organization of the NEXCADE analysis pipeline.

**Figure 2:**
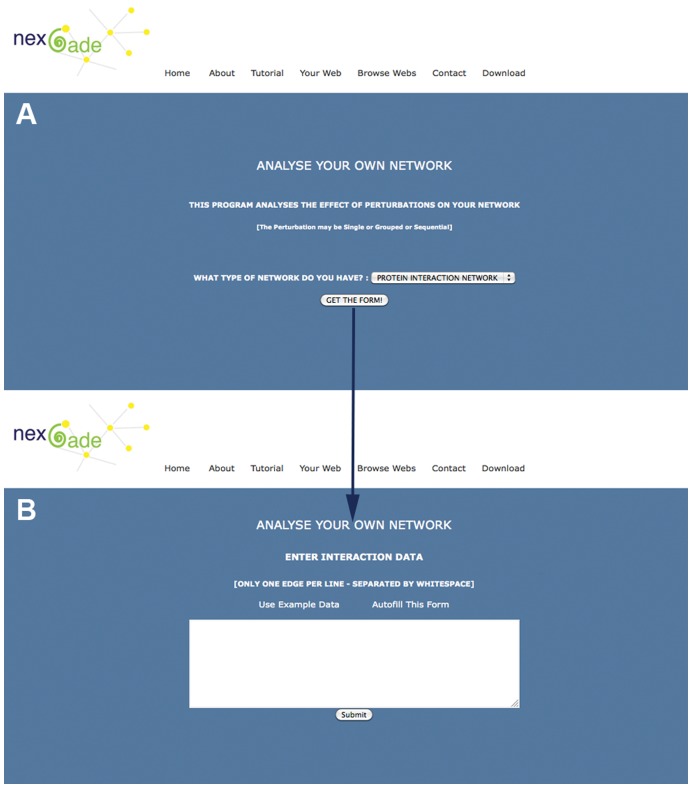
Query submission protocol in the NEXCADE web server. (A) Selection of network type, (B) Data entry form.

In-house Fortran programs and shell scripts were used to streamline and automate the entire analytical process from input data scanning, network preprocessing for topological and statistical properties, and visualization using R source scripts, followed by simulations of single, grouped and sequential perturbations and the comparison and/or plotting of network attributes after simulation. Figure 1 depicts a flowchart for complete pipeline organization of NEXCADE. The program was converted into a web server by incorporating R functions and libraries into CGI on APACHE linux with additional code built in to enable multiple independent instances of the program so that up to 99 users may access the program simultaneously. Figure 2 depicts a schematic overview of the NEXCADE query submission protocol. The source code of the software (Data S1) is being released under the GNU General Public License (v2 1991) (Data S2), as a standalone unix package along with the online web server. It only requires pre-installation of the freely available IGRAPH R CRAN package. Detailed instructions for set up and usage are provided within the package (also in Data S3).

### Example Datasets

An example dataset is provided with the distributed source code for users to test the command line version of the program along with detailed usage instructions and description of the output. In addition, NEXCADE runs were simulated on five publicly available example networks and one original unpublished dataset. These six networks were selected to represent various kinds of biological and social interactions, and to depict the efficacy of NEXCADE in analysis of diverse webs. Each network is given a four-letter reference code (depicted in square brackets below) which is used throughout the text. These six networks include the largest connected component of the *Rattus rattus* protein-protein interaction network [PRAT], the *Arabidopsis thaliana* genetic interaction network [ATHG], and the yeast RNA-protein interaction datasets [YPRN], downloaded from BioGrid (release 2.0.33) [42]. We also include the well studied dolphin community social network [43] [DOLF]. In addition, NEXCADE was applied to two ecological networks including a seed dispersal network [GNIC] from the tropical rainforests of Great Nicobar Island, India (SB Ph.D. thesis) and a pollinator network [MEMM] that represents the structure of a plant-pollinator food web [44]. The outcome of NEXCADE implementation on these networks along with their references is provided on the respective web pages of each network in the Browse Webs section of NEXCADE.

## Results and Discussion

NEXCADE has four major sections for the analysis of a given network, namely, (a) Visualization and Attributes (b) Single Perturbations (c) Grouped Perturbations and (d) Serial Perturbations, each of which enables users to carry out desired simulations and impact analysis. In addition to the analysis component, NEXCADE has two other parts that include ‘Browsing’ example datasets and a ‘Tutorials’ section that illustrates the methodology, ease of operation and the range of situations and outcomes available, by steering users stepwise through the various options. Features of the four individual sections of analysis are described below using case studies from the pre-simulated networks.

### Visualization

**Figure 3:**
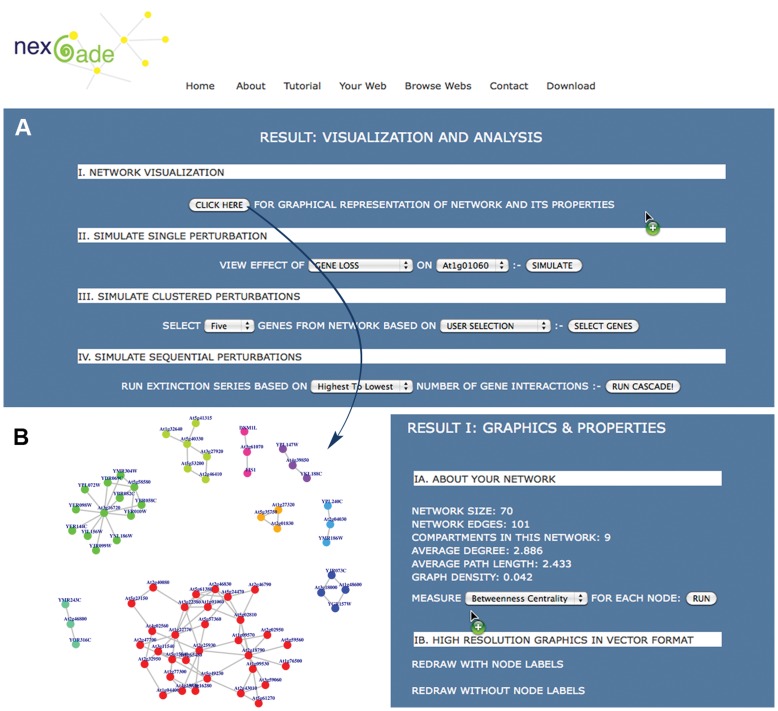
NEXCADE Result & Analyses for a given network. Panel A shows four distinct sections, for input data visualization (Section I) and perturbation analyses (Section II - IV). The lower panel B depicts labeled node visualization for an example dataset with nine disconnected clusters (left) along with topological statistics of the generated network (right).

Figure 3 depicts a screenshot of the visualization page for a given network. Each input network can be visualized as an image containing filled circles connected by lines, the circles representing the nodes of the input network, which may be genes/proteins/species, or any interactor of interest. The lines connecting a pair of nodes represent an interaction between the two nodes. At a single glance, users can have an immediate perception of whether the network is completely connected or fragmented into multiple disconnected clusters, based on node color. As can be seen from this figure, nodes are colored by compartments, such that all nodes that lie in a single connected component of the network have the same color. The nodes in different colors belong to individual disconnected compartments, members of which do not have any interaction with one another in the dataset. Users have the choice to label nodes if required. The visualization section further allows an examination of the basic topological indicators of the network and its components. For example, GNIC is a completely connected network constituted by 812 interactions between 219 species of trees, birds and mammals. It is a highly cohesive network with an average degree of 7.4 and average path length of 3. For each node in the network, NEXCADE measures and displays a sorted list of degree centrality, betweenness centrality, closeness centrality and eigenvector centrality values. A high quality network image can also be downloaded in vector format for obtaining resolution independent figures.

Apart from providing information about these key aspects of network topology, the main purpose of this section of NEXCADE is to assist users in selection of optimal nodes for perturbation i.e whether one would like to maximize the preferential perturbation or minimize it based on the observation that affect of preferential or targeted perturbations is strongly influenced by topological dependencies such a vertex degree. While making rational decisions about what kinds of perturbations to simulate, users can select one or more entities in a single, grouped, random or sequential manner. Perturbations can be simulated on both, interactors as well as interactions in the network, as described below with examples.

### Single Perturbations

**Figure 4:**
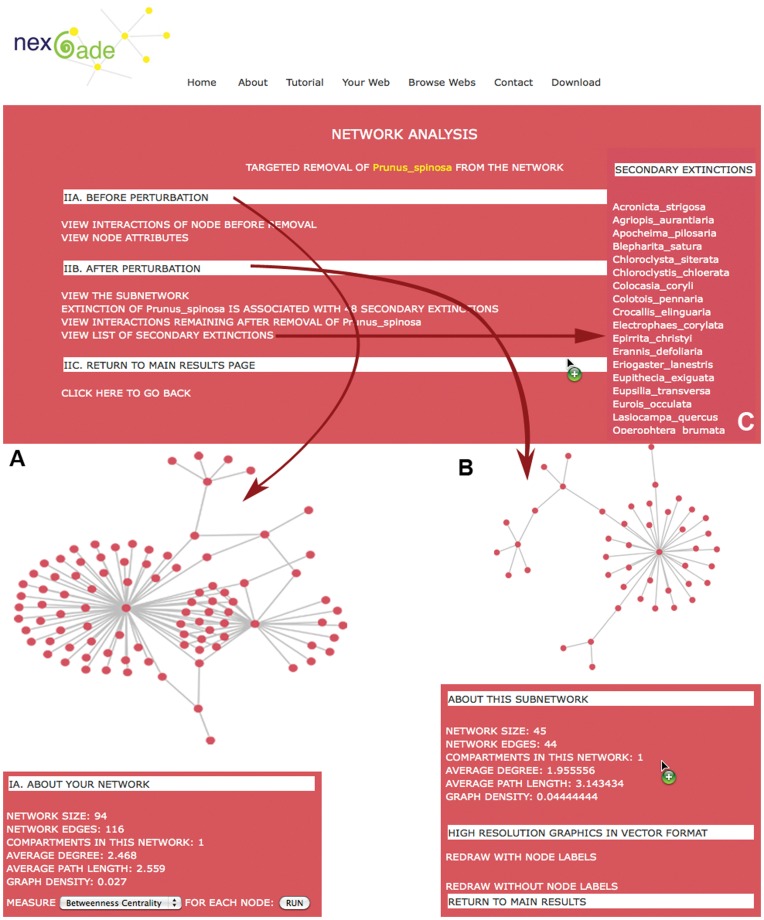
A typical use of NEXCADE for Single Node Perturbation. The network (A) before and (B) after extinction of the species *Prunus spinosa,* are shown. As can be seen, a single perturbation can result in a large number of associated co-extinctions of other affiliate species (Panel C). Users can compare change in network attributes (A with B), and view list of affected nodes, secondary extinctions and remaining interactions in the sub-network. High resolution graphics of networks can be drawn with and without labels.

Depending upon the input dataset, the removal of a single node or edge may represent mutation in a protein, knock-out of a gene, extinction of a species, or even the elimination of a relationship, such as correlated expression between two genes in the dataset. In this section of NEXCADE analysis, users can select any node of their choice and also selectively remove an edge or interaction of the node under consideration and visualize the resulting network. The consequence of such a perturbation can be assessed in terms of changes in the overall node and network level attributes, as shown in Figure 4, as well as in terms of additional perturbations that are either generated or propagated from the initial event. For example, single perturbation analysis shows that removal of the species *Daucus carota,* which is pollinated by several dipteran and hymenopteran insects, would have disastrous consequences for the plant pollinator network MEMM, resulting in at-least ten associated secondary extinctions. Extinction of *Daucus carota* drastically affects network size and density, leading to imminent co-extinctions of many other species in the network. As can be seen in Figure 4, the reduced network has a much smaller size, and higher values of average path length, average degree and density although it retains its single connected character visible in the common color of all nodes in the sub-network. It may be noted that the targeted species has highest degree centralities in the network and removal of such a key node is expected to have disastrous consequences. However, it may not be correct to undermine the relevance of a node just because it has few connections. Sparsely connected nodes are sometimes connectors of critical network modules or functional clusters and their removal can adversely affect the system. Such an affect has been previously observed in the *E.coli* metabolic network, where the role of N-carbamoyl-L-aspartate is vital even though it participates in only three reactions, because it connects the pyrimidine metabolism, to the core metabolism through alanine and aspartate metabolism [23]. A similar affect is observed through NEXCADE upon targeted removal of NTRK1, a tyrosine kinase receptor from the mouse protein-protein interaction network PRAT. This protein has only three reported interactions with proteins that have several links with other proteins, and its removal is not expected to result in any far-reaching affects. However, NTRK1 connects three structural modules within the network and therefore, its removal results in disruption of inter-module communication, fragmenting the network into three distinct subunits.

### Clustered Perturbations

Pairs of genes or proteins often have parallel roles in the cellular milieu, and the removal of such coupled entities can affect the system negatively [24], [25]. An example of this can be seen in NEXCADE, wherein, removal of the protein SLC6A3 alone from the PPI network PRAT, does not damage the network drastically, but when SLCA3 is removed along with another protein ARRB2, the paired perturbation causes the largest connected component of the network to fragment into disconnected clusters. It is clear from the first section for PRAT analysis (Visualization and Attributes) that neither of these proteins is highly connected, but they are both independent connectors between two important modules of the dataset and thus have high structural relevance for the network. Although, removal of either of these is not sufficient to sever inter-module connectivity, it renders the inter-module topology of the network highly susceptible to the next perturbation.

**Figure 5:**
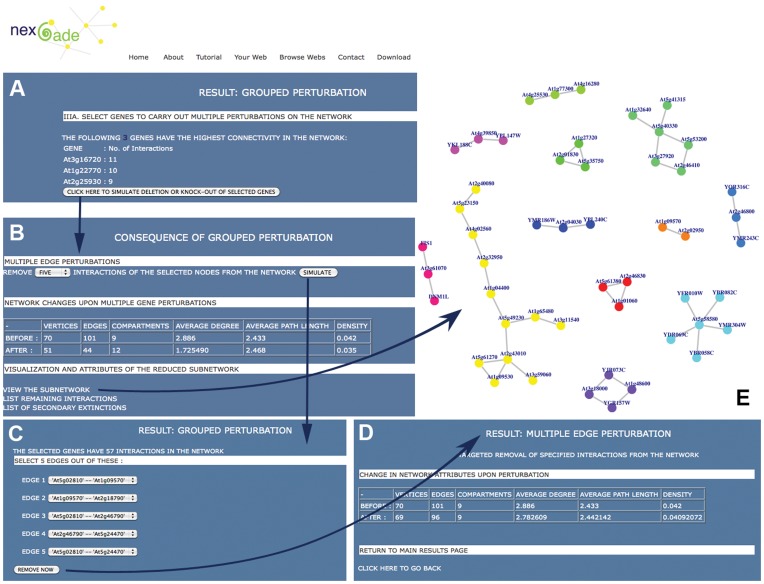
Results of clustered perturbation simulated on the example network shown in **Figure 3**. Clicking section III in Figure 3A enables users to: (A) select a group of target nodes based on degree and (B) analyze the affect of perturbation. Clicking the first option on this page returns Panel (C) - the selection form for simulating clustered edge perturbation on the edges of pre-selected nodes and the resulting sub-network can be analyzed comparatively as shown in (D). As can be seen in panel (E), the reduced sub-network upon clustered node perturbation is much fragmented and smaller, in contrast with the unperturbed network shown in Figure 3B.

The affect of perturbing larger clusters of nodes or edges from a network, rather than in pairs, can also be analyzed in this section of NEXCADE. In the online version, users can specify and target up to nine vertices (or their edges) for inducing clustered perturbations, while the distributed version of the program (Data S1) has no upper limit on the size of the group to be perturbed. For example, Figure 5 shows the affect of removal of three specific genes in the example network ATHG, resulting in fragmentation of the network into several disconnected sub-networks. These genes were selected using NEXCADE by scanning the initial unperturbed network for nodes that have a high betweenness centrality, but not very high degree centralities, demonstrating the ability of the program to assist users in identifying nodes or sets of nodes that may be critical for network sustenance.

### Sequential Perturbations

**Figure 6:**
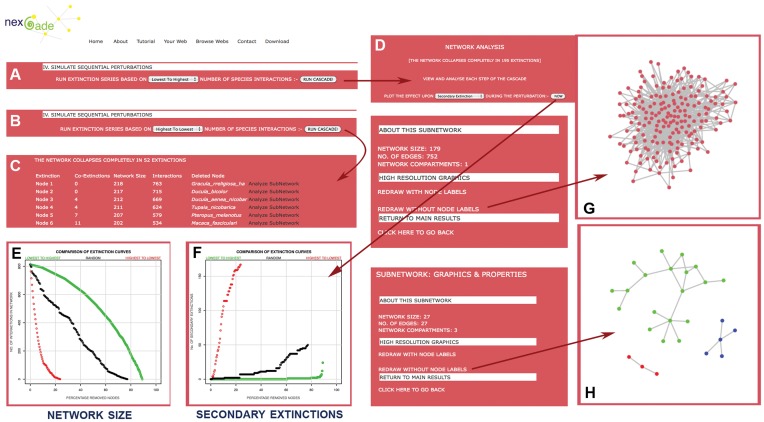
Outcome of random and ordered sequential perturbation on GNIC. Clicking section IV in Figure 3A enables users to simulate three distinct co-extinction curves, namely (A) specialist-first, (B) generalists first, and random cascades. Panels (C) and (D) depict the contrasting network responses between two opposing extinction sequences, resulting in complete collapse at 52 and 195 extinctions respectively. Plots in panels (E) and (F) show this contrasting response in terms of network size and secondary extinctions. When node removals are simulated from the most-linked to least-linked species (red curves), the network collapses much faster, and secondary extinctions begin at a very early stage, as compared to the opposing node loss sequence (green curves). Random co-extinction curves are depicted in Black. Panels (G) and (H) show the status of the network at equivalent number of node removals in the two opposing extinction series.

For cascading or sequential perturbations on the input networks, NEXCADE uses degree centrality as a ranking property to carry out serially ordered perturbations, each involving successive vertex removal. The affect of the perturbation can be analyzed and visualized at each step of the serial extinction cascade as described already. In addition, the overall change in a specific network attribute can be monitored as a function of the percentage removed nodes, throughout the simulation cascade. These curves, called co-extinction curves, are usually curvilinear for real world systems. For example, Figure 6 shows the affect of simulating sequential perturbations on GNIC, in terms of the number of secondary extinctions, as the primary extinctions are carried out. As can be seen from this figure, when targeted extinctions are carried out from the most connected to least connected species, secondary extinctions begin with the deletion of the first node itself. The network quickly disintegrates into several disconnected fragments (within 10% node removals) and undergoes complete collapse within 52 primary extinctions i.e 22% node loss. In contrast, if the preferential extinctions are simulated from the least linked (specialist) to most linked (generalist) species, the network size decreases slowly and secondary extinctions do no occur till almost 90% primary extinctions have occurred. It may be noted that the network does not undergo fragmentation at all and is able to retain its single connected character for more than 90% species removals, revealing the robustness of the network under attack, in terms of its ability to remain stable for much longer lengths of time when perturbed. Figure 6 also shows the corresponding status of the reduced sub-networks after 7% node removals in the two opposing cascades emphasizing the contrasting network response in terms of robustness. As can be seen from this figure, the network is able to withstand the targeted removal of specialists whereas; it is highly susceptible to the removal of generalists. Such a contrasting affect on a system under specific extinction sequences has often been observed in ecological networks and is considered a measure of network robustness. Furthermore, NEXCADE also enables simulation of Random co-extinction curves and comparisons of different perturbation series with each other in terms of their affects on Network topological indicators, as shown in Figure 6. Users have the option to simulate multiple random extinction series on a given network, if necessary. NEXCADE, to our knowledge is the only program that automates the entire targeted extinction cascade approach, thereby enabling users to evaluate and compare network stability.

### Availability, Processing Time & Limitations

NEXCADE is available in two forms, the online and distributed version. One, as an online interactive webserver with a very simple, user-friendly interface and help pages, freely available to the scientific community without any login requirement at http://nipgr.res.in/nexcade.html, that describes the scope of the program along with a tutorial, a feedback form as well as a comprehensive mechanism for testing the program with several different sets of pre-simulated data. The distributed command line version of NEXCADE is a unix tarball containing the source code of the program along with detailed installation and usage instructions, along with an example dataset (Data S1 and S3). The latest version can also be downloaded at http://nipgr.res.in/nexcade_download.html.

A network of about 1000 edges takes approximately four seconds to load on a 3.2 GHz processor with 8 GB RAM. Although NEXCADE can handle networks of any size, processing time may get longer in case multiple parallel sessions of the online program are being run. It may be noted that the webserver is not designed to store datasets for long periods of time, but in case the connection to our servers is lost during a run, or processing becomes extremely time consuming, the results can be accessed after a short period via a five letter code assigned to the user upon data input and visible in the address bar. Further, in case of large networks, the simulations, particularly the compute-intensive co-extinction curves, can be extremely time consuming. Therefore, we recommend the use of the command line version of NEXCADE for large datasets in order to take the load off our servers and for users to store NEXCADE simulation results for as long as desired. The online version is optimally suited to datasets having up to 300 nodes and 1000 interactions and is mainly designed to enable overall assessment of the software and its abilities.

It may be emphasized that one of the implicit assumptions of deletion based perturbation analyses is that the input dataset is sufficiently exhaustive and inclusive. However, this may not always be the case, and unknown dependencies may exist between network components that are not included in the input dataset. Further, limitations of the input data combined with the method (NEXCADE simplifies and reads all input graphs as undirected) may moderate the impact of the analysis and limit the true assessment of disturbances. However, we justify NEXCADE and its applicability to complex system research based on the widely accepted usage of this method of simulating perturbations and the fact that it is a first attempt to automate different kinds of disturbances and prediction of their impact on complex systems.

### Conclusions

In this work, we have given an overview of the rationale, design and implementation of the program NEXCADE that can assist in analysis of perturbations and assessment of the consequences of perturbations on complex systems defined by networks that can be expressed as interconnected matrices of interactions. It enables users to assess the outcome of seemingly minor events such as a random gene mutation or metabolic fluctuation, which once set in motion, may become explosive and in extreme cases, lead to irreversible collapse through a cascade of detrimental affects. Although such analyses are now being used routinely in diverse areas of scientific research, a large number of potential users are unable to use these methods for analysis of their own datasets for lack of mathematical and/or computational skills. NEXCADE bridges this gap in a simple user friendly way. To demonstrate its generality and use in a variety of different scenarios, we have applied NEXCADE to several reported social, ecological and biochemical networks, providing a glimpse of the applications that NEXCADE can be used for. We anticipate that it can have wide-ranging benefits to the scientific community and would facilitate risk assessment and threat based management studies in complex network analysis.

In future versions, we hope to incorporate network loops (e.g. self interactions) and edge weights (e.g. abundance and expression values etc) so as to enable users to analyze the affects of perturbing interaction strengths, thereby emulating ‘knock-downs’ in addition to ‘knock-outs’. We are also currently developing methods to add perturbation specific scores for networks and gain of function perturbations that add new components to an existing network, with user-definable attributes, through an approach similar to the one presented in this paper. Such an extension to NEXCADE would, for example, help to gain insights into biological invasions, and it would also contribute to the development of effective algorithms for more diverse kinds of perturbation analysis, yet to be explored.

## Supporting Information

Data S1 **Compressed/ZIP File Archive.** Contains Unix Tarball of the distributed version of NEXCADE.
(GZ) Click here for additional data file.

Data S2 **OSI License File.** Contains GNU General Public License for use of NEXCADE Software.
(TXT)

Data S3 **NEXCADE Usage.** Contains Complete Software Documentation.
(DOCX)
