# Supplementary material for: NEXCADE: Perturbation Analysis for Complex Networks
Source: PLoS One. 2012 Aug 3;7(8):e41827. doi: 10.1371/journal.pone.0041827 (PMC3411682; doi:10.1371/journal.pone.0041827)
Supplement: Data S3 — NEXCADE Usage. Contains Complete Software Documentation. (DOCX) [file pone.0041827.s003.docx]

TITLE:

NEXCADE: PERTURBATION ANALYSIS OF COMPELX NETWORKS

AUTHORS:

GITANJALI YADAV & SURESH BABU

**ONLINE SUPPLEMENTARY MATERIAL**

PAGES 1 – 17: SOFTWARE DOCUMENTATION

****************************

NEXCADE

COMMAND LINE VERSION

INSTRUCTIONS

****************************

Nexcade is an automated and interactive program for analyzing complex systems that can be conceptualized by networks, and represented as interconnected matrices of interactions. The analytical framework employs a graph theoretical approach for inducing various kinds of perturbations on networks such as extinction cascade simulations, focusing on the changes in topology and connectivity of the network as a function of the perturbation. Perturbations are induced in a user-defined manner followed by analysis of the resulting sub-networks.

The most recent version of the source code for NEXCADE is available at http://nipgr.res.in/nexcade_download.html.

The code is a linux-based tarball containing a computational program that has been developed in the area of network perturbation analysis, based on an in-depth understanding of a large number of ecological, metabolic and gene regulatory networks and their responses to various kinds of perturbations. The program, originally written as a unix code, is also available as an automated online server with the same name in order to be more useful for the scientific community at http://nipgr.res.in/nexcade.html

NEXCADE enables users to :

(a) Induce targeted disturbances into complex systems defined by networks

(b) Visualize and Analyse the consequences of perturbations focusing on the changes in topology and connectivity of the network as a function of the disturbance.

The NEXCADE suite of programs has four major sections for analysis of a given network:

(I) Visualization and Attributes

(II) Simulation of Single Perturbations

(III) Simulation of Grouped Perturbations and

(IV) Simulation of Serial or Cascading Perturbations,

- each of which enables users to carry out desired simulations and impact analysis.

---------------------------------------------------------------------------

TO BEGIN

---------------------------------------------------------------------------

In a Unix-like system, you can start by uncompressing the tarball

(tar -xzvf nexcade-version.tar.gz) and running 'Make_Nexcade' from the nexcade-version directory:

# Enter the Main/Source Directory

cd nexcade-version

# Create a new workspace

./Make_Nexcade

(Type this command in the Source Directory itself)

This command will have to be run each time you need to simulate a new network.

It does the following:

Creates a new sub-directory (name based on TIMESTAMP) inside 'RUNS' dir

Collects all executables in the newly created sub-directory

Puts an example network in the newly created sub-directory for testing.

You can put your own network file into this sub-directory if you wish to use your own data.

Keep in Mind:

Your network file must have the '.ncol' extension in its name

Your network file must be in the two column format described below (DATA INPUT Section).

If all goes well, this will return the following in the source directory:

== ==

NEXCADE IS READY TO USE

TYPE "cd ./RUNS/NEW_SUBDIR_NAME"

& TYPE "./run_nexcade TEST"

== ==

# As shown in the outcome above, Go to the newest sub-directory within RUNS directory

cd ./RUNS/'NAME_OF_NEWEST_DIR_AS_SHOWN_ON_SCREEN'

# Once inside, Run the main script as shown on screen outcome above

./run_nexcade TEST

Here the program will use the example network TEST.ncol

If you have your own network file "YOUR_FILENAME.ncol", then type

./run_nexcade YOUR_FILENAME

Note that the extension '.ncol' must NOT be given to the program

# The 'run_nexcade' script checks the input network and prepares the required files for all three types of perturbation analyses. To carry out any of these kinds of analyses, go to section 'PERURBATION ANALYSES' below.

# To clear space in your area, simply delete the newly created directory after your analysis is complete.

rm -rf `NAME OF NEWEST DIRECTORY'

# To restart the analysis with a new network, return to the Source directory and type the initial command explained above to create a new workspace:

cd ../..

./Make_Nexcade

---------------------------------------------------------------------------

DATA INPUT

---------------------------------------------------------------------------

To run the program in a Linux environment (using a terminal), type

./run_nexcade TEST

where,

TEST.ncol is the input network file

TEST.ncol is the name of the network file that contains the network.

The file must be a list of interactions with the format:

//

node1 node2

node3 node4

. .

. .

. .

//

This represents a network with a link between nodes node1 and node2, another between nodes node3 and node4, and so on. Names of Nodes must be separated by whitespaces or tab. There should be NO spaces within the node names. Avoid complex characters within names like '{([%#!' etc

---------------------------------------------------------------------------

---------------------------------------------------------------------------

---------------------------------------------------------------------------

(I) VISUALIZATION AND ATTRIBUTES

---------------------------------------------------------------------------

After running the first main script

./run_nexcade

on your network, overall network properties are measured, in preparation for subsequent perturbations.

If all goes well, the following line is printed on the screen:

============SCAN COMPLETE!==========

In addition, Several files are created as explained below:

----------------------

DATA.jpg

----------------------

This is a visual image of the network itself.

----------------------

DATA.pdf

----------------------

This is a high resolution image of the network

It also has node labels

----------------------

DATA.details

----------------------

This file contains information on basic network parameters like: Network Size, No. of Interactions/Edges, No. of Compartments, Avr Degree, Avr Path length and Graph density for the network.

----------------------

DATA.stat

----------------------

This file contains information on basic node level properties of the network. For each node, this file contains the values of Degree centrality, Betweenness centrality, Closeness centrality and Eigenvector centrality.

----------------------

DATA.nodes.select

----------------------

This is a list of coded node names for each node in the network. The codes are used for simulating perturbations on user-selected node/s. See the section on 'SINGLE PERTURBATION SIMULATION' for further information and description of how to use this file

----------------------

DATA.edgelist

----------------------

This file contains a coded list of all interactions in the network. The codes are used for simulating perturbations on user-selected edge/s. See the section on 'GROUPED PERTURBATION SIMULATION' for further information and description of how to use this file

----------------------

OTHER FILES

----------------------

Some other files for internal use by the suite are also created by the script.

These include

DATA.degree (the degree centralities of all nodes in the network)

DATA.ncol (the simplified input network file)

DATA.nodes (the names of all nodes in the input network file)

DATA_DEGREEMaxMin_ALL.cascade (For simulating sequential perturbations)

DATA_DEGREEMinMax_ALL.cascade (For simulating sequential perturbations)

---------------------------------------------------------------------------

(II) SINGLE PERTURBATION SIMULATION

---------------------------------------------------------------------------

NEXCADE enables users to :

(a) Induce targetted disturbances into complex systems defined by networks, and

(b) Visualize and Analyse the consequences of perturbations focusing on the changes in topology and connectivity of the network as a function of the disturbance.

---------------------------------------------------------------------------

(IIA) Simulation of Single Node Perturbations

---------------------------------------------------------------------------

#FOR A SPECIFIC NODE

In order to simulate the perturbation of a single node, you must first run the initial two commands described above in the 'TO BEGIN' section. You must be in the same sub-directory where './run_nexcade' was run. Thereafter:

1. Select the node you wish to perturb [from file 'DATA.nodes']

2. Get the numerical code 'N' for the selected node from file 'DATA.nodes.select'

3. Run the following command

'./run_single_perturbation NODE N'

Note that this script requires TWO input parameters:

The First is the word 'NODE' (all caps) specifying that a NODE has to be perturbed.

The Second is the integer 'N' that signifies a specific node in the network

For example:

'./run_single_perturbation NODE 21'

Note that '21' signifies the species 'Turdus pilaris' in the file 'DATA.select.nodes'

More Examples:

'./run_single_perturbation NODE 2'

'./run_single_perturbation NODE 16'

#If all goes well, the following line is printed on the screen:

======== RUN COMPLETE! =======

#In addition, Several files are created as explained below:

----------------------

node_N.iname

----------------------

This file contains the name of the perturbed node.

----------------------

node_N.attr

----------------------

This file contains the node attributes of the perturbed node BEFORE perturbation. These include the four basic centralities; Degree, Betweenness, Closeness and Evcent.

----------------------

node_N.int

----------------------

This file lists the other nodes in the network that were interacting with the perturbed node BEFORE perturbation. The same information is provided in network format in the file node_N.ncol. You can use this file for starting a new analysis.

----------------------

node_N.details

----------------------

This file contains the network attributes of the subnetwork remaining AFTER perturbation. These include Subnetwork Size, its no. of edges, total compartments, average node degree, avr. path length and graph density.

----------------------

node_N.jpg

----------------------

This is a visual image of the network AFTER the perturbation. There is also a high resolution image in pdf format "DATA.pdf"

----------------------

node_N.secex

----------------------

This file lists the names of other network nodes that are adversely affected as a result of the induced perturbation of node N. Node names in this file, if any, represent the additional nodes that get go extinct upon perturbation of the node N. Note that this file may sometimes be empty, signifying that no other nodes have been lost.

----------------------

node_N_rem.ncol

----------------------

This file represents the subnetwork remaining AFTER the perturbation, in the ncol format. You can use this file as input to begin a new NEXCADE run.

----------------------

Other Points to Remember

----------------------

N must always be an integer.

N cannot exceed the total network size.

Only one value of N can be given at a time.

The script can be used to preturb each node in the network one after the other.

Each set of outcomes will be named after the integer code of the perturbed node.

---------------------------------------------------------------------------

(IIB) Simulation of Random Single Node Perturbations

---------------------------------------------------------------------------

An interesting approach is to see how the network reacts to the random removal of any one node at the time. For this, all nodes need to be taken out, one at the time and network properties need to be calculated and plotted across the removal of all the individual nodes while the network size remains constant as complete network minus one.

These curves can than be compared across networks to assess how each of the networks reacts to the removal of a random node.

NEXCADE enables this approach as follows:

1. Run the command

'./run_single_perturbation_all

This simulation can take some time, depending upon the number of nodes in the network.

At the end, the following files will be produced:

----------------------

random_single.out

----------------------

This is a file containing seven fundamental network attributes after each node is removed

and before it is put back into the network. Each Row corresponds to one node, that is

removed at that level of perturbation.

It contains the following columns:

NAME: Name of the Node Removed

SECEX: No. of Secondary Extinctions after Perturbation

SIZE: Size of the Network after Perturbation

EDGES: Interactions Remaining in the Network after Simulation

COMP: No. of Compartments

AVR_DEG: Average Degree of the Network after Perturbation

AVR_PL: Average Pathlength of the Network after Perturbation

DENSITY: Graph Density

----------------------

random_single.jpg

----------------------

This is a plot of the change in three network attributes

during the random single perturbations simulated above.

These three attributes are :

1.No. of Compartments

2. Avergare Degree

3. No.of Seconday Extinctions

There is also a high resolution imgae in pdf format "random_single.pdf"

---------------------------------------------------------------------------

(IIB) Simulation of Single Edge Perturbations

---------------------------------------------------------------------------

In order to simulate the perturbation of a single edge or interaction:

1. Select the edge you wish to perturb [from file 'DATA.edgelist']

2. Get the numerical code 'N' for the selected edge from file 'DATA.edgelist'

3. Run the follwoing command

'./run_single_perturbation EDGE N'

As before, this script requires TWO input parameters:

The First is the word 'EDGE' (all caps) specifying that an EDGE has to be perturbed.

The Second is the integer 'N' that signifies a specific edge in the network

For example:

The node 'Rubus.sp' in the TEST network has a total of five interactions, one of which is with 'Parus_Palustris'. Suppose you need to remove this interaction, note its code given in brackets on the left: It is the integer '8'. Thus, to perturb this edge, type:

'./run_single_perturbation EDGE 8'

Other Examples:

'./run_single_perturbation EDGE 0'

'./run_single_perturbation EDGE 44'

#If all goes well, the following line is printed on the screen:

======== RUN COMPLETE! =======

Once again, a number of files are produced to represent the sub network remaining after edge perturbation.

These are:

edge_8.details ( Network properties of the subnetwork after perturbation)

edge_8.jpg (Subnetwork Visualization - image)

edge_8.nodes (Names of all nodes remaining in the sunnetwork)

edge_8.pdf (Subnetwork Visualization - High Resolution Image in PDF format)

edge_8.secex (Secondary Extinctions, if any, associated with the perturbation)

----------------------

Other Points to Remember:

----------------------

N must always be an integer.

N cannot exceed the total number of interactions in the network.

Only one value of N can be given at one time.

The script can be used to preturb each edge in the network one after the other.

Each set of outcomes will be named after the integer code of the perturbed edge.

All files will remain in the same sub-directory (under RUNS)

---------------------------------------------------------------------------

(III) GROUPED OR CLUSTERED PERTURBATION SIMULATION

---------------------------------------------------------------------------

Pairs of genes or proteins often have parallel roles in the cellular milieu, and the effects of removal of such coupled entities can affect the system negatively. Users can select any two nodes or edges from the network to simulate paired perturbations. In addition, the effect of perturbing larger clusters of nodes or edges from a network, rather than just pairs, can also be analyzed in this section of NEXCADE, by specifying any number of vertices (or edges).

The script 'run_group_perturbation' is used for this simulation with THREE parameters:

1. The number of entities to perturb. (From 2 onwards)

2. The selection of entities to perturb. (User specified or Most-Connected or Least-Connected)

3. The type of entity to perturb. ('NODE' or 'EDGE')

FOR NODES:

---------------------------------------------------------------------------

(IIIA) Simulation of Multiple Node Perturbations

---------------------------------------------------------------------------

In order to simulate the perturbation of a group of nodes, You must be in the same sub-directory where initial 'Run_nexcade' script was run.

For Example:

If you wish to perturb the 7 MOST connected nodes from the network, type:

'./run_group_perturbation 7 MAX NODE

Other Examples:

'./run_group_perturbation 5 MIN NODE ( To Perturb Five Least conencted Nodes)

'./run_group_perturbation 10 USR NODE ( To Perturb Ten User Specified Nodes)

[For the 'USR' option, you must SPECIFIY the list of nodes to be perturbed. This list MUST be in a file named 'group.input'. You can select integer codes of the nodes you wish to perturb from file 'DATA.nodes.select' and enter into this file, one per line. The file must be in the same sub-directory where the initial 'run_nexcade' script was run. For example, the file 'group.input' may contain five lines as follows:

---------//

12

2

14

21

7

---------//

To perturb the five nodes specified in this file, simply type:

'./run_group_perturbation 5 USR NODE

]

#If all goes well, the following line is printed on the screen:

======== RUN COMPLETE! =======

Once again, a number of files are produced to represent the subnetwork remaining after group perturbation.

The nomenclature of these files is such that, depending upon the mode of perturbation, the output file names will be of the format 'nodes_NXXX.xyz' in the prefix, where N is the number of nodes perturbed and XXX is the mode of node selection.

For example:

"nodes_5MAX.xyz" format: If 5 MAX ( most ) connected nodes have been perturbed.

"nodes_9MIN.xyz" format: If 9 MIN ( least) connected nodes have been perturbed.

"nodes_7USR.xyz" format: If 7 USR ( user selected) nodes have been perturbed.

(.xyz is the file extension and may differ based on the kind of file produced)

In each case, the following set of files is returned:

nodes_NXXX.names (Names of the nodes selected for perturbation)

nodes_NXXX.ncol (List of interactions of selected nodes BEFORE perturbation)

nodes_NXXX.details (Network properties of the subnetwork AFTER perturbation)

nodes_NXXX.stat (Node level Subnetwork Statistics AFTER perturbation)

nodes_NXXX.jpg (Subnetwork Visualization AFTER perturbation - image)

nodes_NXXX.pdf (Subnetwork Visualization - High Resolution image- PDF format)

nodes_NXXX.secex (Secondary Extinctions, if any, associated with the perturbation)

nodes_NXXX_rem.ncol(Interactions in the subnetwork - AFTER perturbation)

FOR EDGES:

---------------------------------------------------------------------------

(IIIB) Simulation of Multiple Edge Perturbations

---------------------------------------------------------------------------

In order to simulate the perturbation of a group of edges, You must be in the same sub-directory where initial 'Run_nexcade' script was run. You must ALWAYS specify the list of edges to be perturbed. This list MUST be in a file named 'group.input'. Select integer codes of the edges you wish to perturb from file 'DATA.edgelist'. Enter each edge-code into the file, 'group.input' one per line. The file must be in the same sub-directory where the main script was run initially.

For example, the file 'group.input' may contain five lines as follows:

---------//

32

6

14

1

47

---------//

To perturb the five edges specified in this file, simply type:

'./run_group_perturbation 5 USR EDGE

]

#If all goes well, the following line is printed on the screen:

======== RUN COMPLETE! =======

As with node perturbation, a number of files are produced to represent the subnetwork remaining after group perturbation. The nomenclature of these files is of the format 'edges_x.xyz', where xyz is the suffix (or file extension).

In each case, the following set of files is returned:

edges_x.nodes (Names of the nodes remaining in the subnetwork AFTER perturbation)

edges_x.details (Network properties of the subnetwork AFTER perturbation)

edges_x.secex (Secondary Extinctions, if any, associated with the perturbation)

---------------------------------------------------------------------------

(IV) SEQUENTIAL PERTURBATION SIMULATION

---------------------------------------------------------------------------

As described earlier, the fourth and last section in NEXCADE allows users to simulate complete cascades of perturbation on the system of interest. The script runs two opposite sequences of node-removals to carry out serial extinctions, based on DEGREE centrality, or the connectivity attribute.

The first is the 'Highest To Lowest' node based sequential cascade, which simulates removal of every node from the network beginning with the node that has the highest

(MAX) connectivity, to the node that has the lowest (MIN) connectivity in th network. The code for simulation is 'MAXMIN'.

The second is the 'Lowest To Highest' node based sequential cascade, which simulates sequential removal of nodes from the network in the exact reverse order.

The code for simulations is 'MINMAX'

In addition, the script also runs a RANDOM Extinction Cascade, to compare with

the previous two sequentially ordered cascades.

After completing the three cascades, the script analyses and plots the changes observed in

critical network level properties during the entire duration of a cascade.

Plots can be visualized separately for each cascade, or in pairs, or even for all three i

series together, as explained below..

In order to simulate complete cascades of extinction, simply type:

'./run_cascades

within the sub-directory where initial 'Run_nexcade' script was run. The script uses the two '.cascade' files generated when 'Run_nexcade' was run. This script does not require any external parameters.

#If all goes well, the following lines are printed on screen:

===== SIMULATING MINMAX CASCADE ===

THE NETWORK COLLAPSES COMPLETELY IN 'N' SEQUENTIAL PERTURBATIONS

===== CASCADE COMPLETE! ===========

===== SIMULATING MAXMIN CASCADE ===

THE NETWORK COLLAPSES COMPLETELY IN M' SEQUENTIAL PERTURBATIONS

===== CASCADE COMPLETE! ===========

===== SIMULATING RANDOM CASCADE ===

THE NETWORK COLLAPSES COMPLETELY IN 'K' SEQUENTIAL PERTURBATIONS

===== CASCADE COMPLETE! ===========

===== NOW PLOTTING SIMULATION RESULTS ===========

===== SINGLE SERIES PLOTS COMPLETE! ========================

===== PAIRWISE SERIES PLOTS COMPLETE! ======================

===== ALL SERIES PLOTS COMPLETE! ========================

As can be seen above, users can directly read the outcome of cascading perturbation on screen. N,K and M represent the number of consecutive perturbations required to collapse the original network in the two opposing sequences.

A number of files are produced for each extinction cascade to enable further analysis:

The names of these files contain the term 'MinMax' or 'MaxMin'or 'Random' depending upon the cascade sequence.

For each cascade, the following set of files is returned:

Note:

xxxxxx below refers to the cascade type - 'MinMax' or 'MaxMin' or 'Random'.

NNNN below refers to the Nth extinction in the respective sequence.

----------------------

DATA_DEGREExxxxxx_ALL.result

----------------------

This file contains the Tabulated outcome of each successive perturbation within each cascade.

Each row corresponds to one single node perturbation

The file has nine columns of data:

1. Network Name: The name of the network file at each successive perturbation

2. S.No The serial number of the current pertrubation ( for that row)

3. Pri_Ext Cumulative No. of Induced Perturbations at current perturbation level

4. Tot_Ext Cumulative No. of Total (Pri + Secondary) perturbations at current level

5. Sec_Ext Secondary Extinctions associated with the current perturbation

6. Size Network Size after current perturbation

7. Rem_Int No. of Interactions Remaining in the network at current perturbation

8. Lost_Int No. of Interactions lost from original network at current level

9. NodeName The name of the Node that has been perturbed at the current level

----------------------

DATA_DEGREExxxxxx_ALL_NNNN.ncol

----------------------

There will be as many files of this format as the number of nodes.

Each such file represents the subnetwork after the NNNNth consecutive perturbation.

It constains a series of interactions. You can use this file for starting a new analysis.

----------------------

DATA_DEGREExxxxxx_ALL_NNNN.ncol.extinct

----------------------

This file will be generated if there are any secondary extinctions associated with the

NNNNth consecutive node perturbation. Many such files will be generated after a cascade

simulation. The name of the file includes the cascade sequence as well as the perturbation No.

----------------------

PLOT/GRAPH IMAGE FILES

----------------------

After the cascade simulation, a number of image files are generated.

EACH POINT ON THE GRAPHS PLOTTED AFTER THE SIMULATION REPRESENTS THE SUBNETWORK AT A GIVEN LEVEL OF PERTURBATION [SHOWN ON THE X-axis]. THE Y-axis SHOWS A SPECIFIC NETWORK ATTRIBUTE FOR EACH SUBNETWORK ALONG THE CASCADE DURING THE SIMULATION.

Following image files are generated for each cascade:

DEGREE_xxxxxx_size.jpg (Plot of change in 'SIZE' of each successive network during the simulation)

DEGREE_xxxxxx_remi.jpg (Plot of change in 'No. of INTERACTIONS' during the simulation)

DEGREE_xxxxxx_secx.jpg (Plot of change in 'SECONDARY EXTINCTIONS' during the simulation)

DEGREE_xxxxxx_totx.jpg (Plot of change in 'TOTAL CUMULATIVE EXTINCTIONS' during the simulation)

where:

xxxxxx refers to the cascade type - 'MinMax' or 'MaxMin'.

NNNN refers to the Nth extinction in the respective sequence.

Following image files are generated for each pair of cascades:

DEGREE_Pair_xxxxxx_yyyyyy_zzzz.jpg

(Plot of change in attribute 'zzzz' for co-extinction series xxxxxx and yyyyyy)

DEGREE_All_zzzz.jpg

(Plot of change in attribute 'zzzz' for all three co-extinction series)

----------------------

Other Points to Remember

----------------------

To run a new Random series of extinctions, and plot its results with existing ones,

run the command:

./run_random_cascade

If all goes well, the screen will return the following:

===== SIMULATING NEW RANDOM CASCADE ===

THE NETWORK COLLAPSES COMPLETELY IN 19 SEQUENTIAL PERTURBATIONS

===== CASCADE COMPLETE! ===========

===== NOW PLOTTING SIMULATION RESULTS ===========

===== SINGLE SERIES PLOTS COMPLETE! ========================

===== PAIRWISE SERIES PLOTS COMPLETE! ======================

===== ALL SERIES PLOTS COMPLETE! ========================

As before, a set of files for 'Random' series will be written again.

It will also return:

4 plots for the Random Series,

8 plots for Paired series,

and

4 plots for All three series together

(With the same nomenclature as described above)
